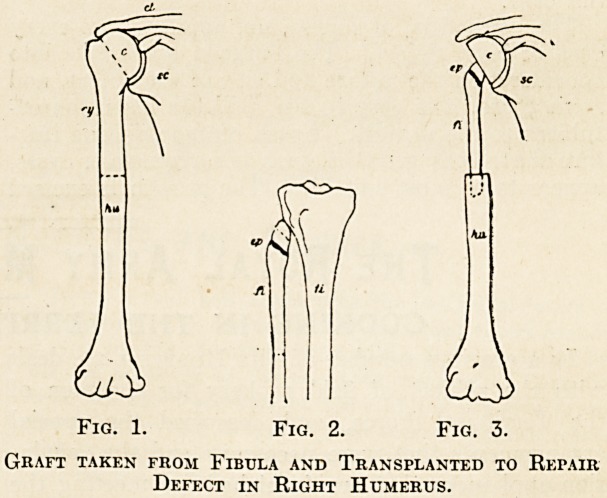# Bone Transplantation
**Der gegenwärtige Stand unserer klinischen Erfahrungen über die Transplantation lebenden menschlichen Knochens.* Von Dr. Eduard Streissler. Tübingen, 1910.


**Published:** 1911-08-05

**Authors:** 


### SPECIAL ARTICLE.

### BONE TRANSPLANTATION.*

Since the brilliant results obtained by Lexer in the transplantation of joints, it is highly desirable to have some collated evidence of the success or otherwise of the various attempts which have been made to transplant portions of bone. When we speak of transplantation we mean, of course, an operative procedure undertaken with the object of remedying some definite pathological condition; not the laboratory experiments which have attracted so much attention in America, and which have not been tested by clinical experience. The general practitioner, no less than the surgeon, wishes to know, in certain cases, whether this new operation of transplanting a whole or a portion of a bone is likely to be of use to his patient. He wishes to know what are the probabilities in the way of risk, prognosis, ultimate and immediate after-effects. Hitherto it has been very difficult to obtain reliable information. Cases have been published, it is true, but in journals which are not readily available, and no practical attempt has been made to gather together the evidence and discuss the pros and cons of the new method from the vantage-point of a large personal experience.

### The Brilliant Work at the Graz Hospital.

That Streissler has been able to do so, in the admirable book which we have before us—a monograph that fully reveals the painstaking care, energy, and accurate workmanship that characterises everything emanating from Professor von Hacker's clinic—is due largely to the fact that he is himself one of the foremost authorities on the subject with which he deals, and has made a thorough study of the literature, besides working at the practical side of the question for many years. Here he publishes twenty-nine cases of direct bone transplantation, every one interesting when standing by itself, but even more interesting when gauged by the results obtained in other cases, and he has added to the list 191 cases recorded in the literature. To surgeons, and especially to orthopædic surgeons, this monograph is of the greatest practical value, and deserves to be more widely known than it is at present. We can here only give a brief abstract of the findings contained in the book; in justice to the author his full descriptions and the histories on which these conclusions are based, together with the experimental work carried out by him at Graz, ought to be read in the original.

### Streissler's Case of Repair of the Humerus.

Bone transplantation, to replace or repair injured bony structures, is not an entirely modern procedure. Jakimowitsch as early as 1881 gave a summary of the theoretical arguments for and against such an operation, and in 1891 Kummel replaced a defect in a patient's radius by a piece of tibia taken from an ox. Since then various cases, some most complicated, have been reported. Some idea of the extent of the operation in certain cases may be obtained from the attached diagrams, which represent the transplantation of a piece of fibula, taken from the same patient, to restore a large defect in the humerus.

The patient was a young man who suffered from a large bony cyst in the upper portion of the right humerus. The cyst was thoroughly removed, the bone being taken away as far as shown in the first diagram, leaving a gap of 11 cm. in length. A piece of bone was then taken from the fibula on the same side, 12 cm. in length, as shown in the second diagram, care being taken to strip off the periosteum and to leave the epiphysis of the head of the bone uninjured, so that there remained a tube of periosteum between the excised ends of the bone. The piece of bone taken away was then pointed roughly at both ends and transplanted into the medullary cavity of the humerus ends, so as to fill the gap (third diagram). Both wounds healed by first intention, and the $x$-ray photographs included in the report show that three months after

Fig. 1. Fig. 2. Fig. 3.

Graft taken from Fibula and Transplanted to Repair Defect in Right Humerus.

the operation the patient had practically a normal arm. Six months later the condition was still excellent; the boy could do fairly heavy work with the arm, and the $x$-ray photographs showed that the bony gaps in both limbs had filled in well.

Several other examples equally striking are cited by Dr. Streissler, and the excellent skiagrams reproduced give very valuable information regarding the progress and fate of the transplanted pieces. Particularly interesting is the nineteenth case, in which part of the neck of the femur was resected for a large bony cyst and replaced by a piece of bone taken from the ilium. This plastic operation was so excellently done, and the after treatment was so conscientious and careful, that the results obtained are really magnificent when one realises the extent of the gap and the difficulties that attended the operation.

* *Der gegenwärtige Stand unserer klinischen Erfahrungen über die Transplantation lebenden menschlichen Knochens.* Von Dr. Eduard Streissler. Tübingen, 1910.

The Technique.

Dr. Streissler takes his bone grafts preferably from the tibia or fibula. These bones are easy of access to the surgeon, and the comparative safety with which they can be chiselled makes them the sites of election for the second operation. A point on which he lays great stress is the necessity for the most scrupulous asepsis; once the wound is infected the chances of obtaining union are very much limited. He fills his gaps ingeniously, each case being treated on its merits and according to the individual conditions present. *X*-ray examinations are taken as often as possible to show the position and the condition of the grafts when they have been transplanted. In the case of the long bones the graft is usually securely immobilised by wiring or aluminium plating; as additional precaution a correctly adjusted plaster casing is put on the limb, and, when necessary, extension is made. The after-treatment needs great care and attention on the part of everyone concerned, but the admirable results obtained at the Graz clinic seem to justify the operation being undertaken much more frequently than is at present the case.

The method is, of course, not applicable to every case of bony defect. The surgeon must take into consideration the nature and site of the defect, and some cases, it is easy to see, will tax his resourcefulness to the utmost. Cases of spontaneous fracture due to bony growths, such as sarcomata or cysts, appear to be most suitable. The growth is excised well away from its centre, and the gap is filled in by a graft taken as advised. Cases in which such a procedure in the interests of the patient is advisable are necessarily rare, and some discrimination must be shown in dealing with malignant growths. The indications where transplanting is justifiable will, however, be easily understood by surgeons who have to deal with such cases. Cases of deformity, owing to bony defect, through fracture or osteomyelitis, seem particularly suitable for this operation. The fate of the transplanted bone is fully discussed in the book. Elucidative illustrations in colour are given, showing the microscopical changes which are noticed, and these are among the most interesting in the book. Decalcified bone-grafts do not seem to be so useful as living grafts, with or without periosteum, and it is always preferable to take these grafts, wherever possible, from the patients themselves. Grafts denuded of their periosteum are liable to be absorbed, but are useful in some cases because they form a bridge, as it were, round about which the new bone grows. Grafts taken from animals, even when retaining their periosteum, are not so good as those taken from the patients themselves; they gradually get absorbed, and their periosteum appears to lose its power of proliferating in the altered surroundings.

Dr. Streissler's work is made doubly valuable by a very full bibliography which is appended, and is certainly one of the most interesting monographs on operative procedure which has appeared during the past year, and one upon which he and his assistants are heartily to be congratulated.

## Figures and Tables

**Fig. 1. Fig. 2. Fig. 3. f1:**